# Carbon Fiber/PLA Recycled Composite

**DOI:** 10.3390/polym14112194

**Published:** 2022-05-28

**Authors:** Salem Al Zahmi, Saif Alhammadi, Amged ElHassan, Waleed Ahmed

**Affiliations:** 1 Department of Chemical & Petroleum Engineering, United Arab Emirates University, Al Ain P.O. Box 15551, United Arab Emirates; 201770302@uaeu.ac.ae; 2 National Water and Energy Center, United Arab Emirates University, Al Ain P.O. Box 15551, United Arab Emirates; 3 Department of Mechanical and Aerospace Engineering, United Arab Emirates University, Al Ain P.O. Box 15551, United Arab Emirates; 201450104@uaeu.ac.ae; 4 Engineering Requirements Unit, United Arab Emirates University, Al Ain P.O. Box 15551, United Arab Emirates; w.ahmed@uaeu.ac.ae

**Keywords:** carbon fiber-reinforced polymer composites, pure carbon fiber, mechanical treatment, tensile strength

## Abstract

Due exceptional properties such as its high-temperature resistance, mechanical characteristics, and relatively lower price, the demand for carbon fiber has been increasing over the past years. The widespread use of carbon-fiber-reinforced polymers or plastics (CFRP) has attracted many industries. However, on the other hand, the increasing demand for carbon fibers has created a waste recycling problem that must be overcome. In this context, increasing plastic waste from the new 3D printing technology has been increased, contributing to a greater need for recycling efforts. This research aims to produce a recycled composite made from different carbon fiber leftover resources to reinforce the increasing waste of Polylactic acid (PLA) as a promising solution to the growing demand for both materials. Two types of leftover carbon fiber waste from domestic industries are handled: carbon fiber waste (CF) and carbon fiber-reinforced composite (CFRP). Two strategies are adopted to produce the recycled composite material, mixing PLA waste with CF one time and with CFRP the second time. The recycled composites are tested under tensile test conditions to investigate the impact of the waste carbon reinforcement on PLA properties. Additionally, thermogravimetric analysis (TGA), X-ray diffraction (XRD), and Fourier-transformed infrared spectroscopy (FTIR) is carried out on composites to study their thermal properties.

## 1. Introduction

Plastic consumption has increased rapidly in several fields. As a result, waste generation has increased during the last century. The generation of waste is not only a challenge for humanity but also a major issue for the stability of the whole ecosystem [1]. Therefore, researchers are encouraged to work on environmental solutions to the problem of collecting and increasing waste of polymeric materials to protect the earth’s natural resources and to develop a sustainable society. Several suggestions have arisen in response to studies by researchers including curbing the use of plastics, applying bio-degradable plastic materials, shifting towards non-plastic materials, etc., according to the needs of the society [2]. Proposals for a sudden shift to remove plastics from the world, however, is criticized by many sectors, including products and services, because a drastic shift may cause economic instability in the world due to the linkage of a wide variety of economic and social strings with the plastic industry [3]. 

Therefore, the application of biodegradable plastics has been presented as the most viable solution according to the current situation by both researchers and industrialists [4]. This nod by the main stakeholders and interest of the public towards the conservation of a healthy environment with less plastic usage in daily life has increased the research on biodegradable polymers [5]. 

### 1.1. Polylactide (PLA)

**Figure 1:**
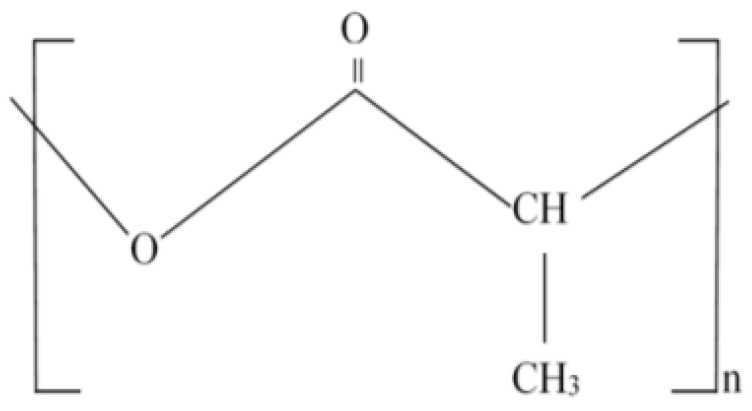
Chemical Structure of Polylactide Acid.

Polylactide (PLA), one of the bio-based polymers used as shown in Figure 1, is a biodegradable polymer utilized in a wide range of applications including automation, packaging, and 3D printing. Because it is easily available and convenient, polylactic acid PLA is an ideal candidate for a polymeric material; yet it lacks thermal and mechanical stability. PLA is predicted to break down at a temperature of around 400 degrees Celsius. However, because of its limited thermal stability, recycling the material and extracting valuable components from the matrices is time-consuming [6].

Polylactic acid (PLA) is an aliphatic polyester. Carothers DuPont discovered PLA in 1932 by heating lactic acid under a vacuum. PLA has piqued the interest of researchers over the last two decades due to its superior processability and characteristics when compared to other biodegradable polymers. Pre-preg is a composite material composed of “pre-impregnated” fibers and partly cured polymer matrices, including epoxy or epoxy resins, or a thermoplastic combined with fluid elastomers or resin [7,8]. PLA has been widely electrospun—pure PLA, PLA blends, and their nanocomposites created with metals, metal oxides, and carbon nanotubes—to control its functionality concerning end purposes, making it superior to petroleum-based polymers such as polyethylene (PE), polypropylene (PP), polystyrene (PS), polycarbonate (PC), and polyethylene terephthalate (PET) [9]. Due to the substantially inadequate mechanical performance of pure thermoplastic material like PLA [10], the mechanical properties can be improved by adding reinforcement material such as continuous carbon fiber (CCF) to the thermoplastic matrix to form a continuous carbon fiber reinforced polymer composite (CCFRPC), which could be used in different engineering applications, such as 3D printing. The reinforcement of natural fibers with biopolymers is an efficient technique to develop composites that are fully biodegradable [11], by incorporation of various percentages of untreated and alkali-treated Coir Fibers (CF) and pineapple leaf fibers (PALF) in PLA biocomposites and characterizations of flexural, morphological, and dynamic mechanical properties that will provide attractive consideration of these hybrid biocomposites for various lightweight uses in a broad selection of industrial applications. Unidirectional flax fabrics were used to reinforce poly(lactic acid) (PLA). Flax/PLA composites were produced by thermo-compression using as-received flax fibers and titanium dioxide (TiO_2_) coated flax fibers [12], to investigate the effect of annealing temperature and time, under quiescent or mechanical stress conditions, on the microstructure, interfacial adhesion, crystallization, and mechanical properties of composites.

The development of creative ways for generating sustainable solutions for manufacturing items with high durability, longer shelf-life, and quality retention after recycling is required by the goal of the plastic economy. Additionally, the effective recycling of the plastics and the multi-layer plastics is considered challenging due to their composite nature [13]. For such cases, mechanical recycling is more resource-efficient; however, it is limited to exposure to harmful substances included in the recycling process as compared to incineration and chemical recycling. To reuse the recycled materials, 3D printing can be used [14].

Commercial PLA degrades slowly under natural settings because its continued existence may harm the ecosystem. Moreover, discarding the PLA due to slow natural degradation is not a wise choice due to the loss of many useful compounds such as the hydroxyl group [15]. Therefore, customized settings of the important parameters in this regard are necessary for the degradation of PLA [16]. Furthermore, throwing out PLA can result in the loss of essential components such as hydroxyl groups. As a result, recycling PLA is crucial to limiting the use of renewable resources for such monomers [17].

PLA recycling is greatly desired because it may be used to generate environmental compatibilizers that can be utilized to improve composite products made from PLA, preserving the material’s eco-friendliness. PLA-based composites with natural fiber have numerous applications due to their easy processing, low toxicity, high mechanical strength, etc., making the need for sustainable recycling of these materials an effective need of a sustainable environment [17]. Furthermore, because PLA is substantially heavier than other commodity polymers (e.g., high-density polyethylene (HDPE)), and low-density polyethylene (LDPE), it can be easily separated based on density, making polymer retention very useful [18].

### 1.2. Carbon Fiber Reinforced Polymers (CFRP)

**Figure 2:**
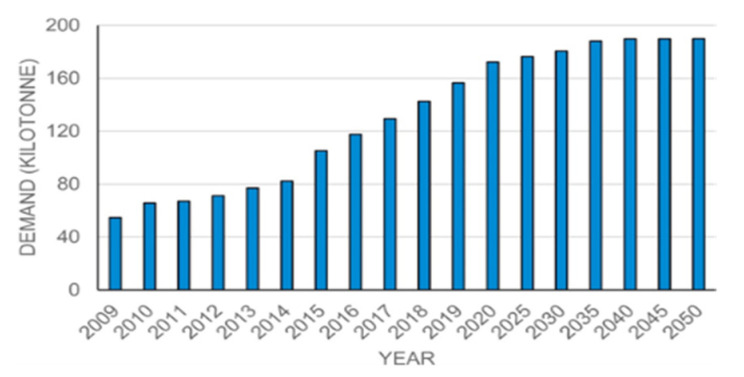
Increasing Carbon Fiber demand over the Years [11].

Carbon fiber reinforced polymers or plastics (CFRP) are gaining popularity owing to their wide range of applications in industries such as aviation, military, vehicle manufacturing, transportation, architecture, sports industries, and medicine [19,20]. Carbon fibers were initially created in the 1960s, and by 2006, almost 27,000 tons of carbon fibers were being produced worldwide, with that figure expected to rise to 140,000 tons by 2020 [21]. Carbon fiber demand has increased by 15% each year over the last few years, as shown in Figure 2. Therefore, the increasing demand for Carbon Fiber Reinforced Polymers (CERP) is also considered necessary due to the increasing consumption of plastic, which leads to increased plastic waste, thereby resulting in the growing demand for better and more efficient recycling [22,23,24,25,26].

Furthermore, no expensive equipment or consumables are needed, and low-skilled labor is sufficient to manufacture the parts, further reducing costs [27,28]. As a result, due to high material and manufacturing costs, upcycling waste core material has the potential to expose new products, processes, and markets for composites that were formerly unattainable to virgin composites. To counter this, the extrusion process is presented as a viable solution that assists in the degradation of polythene, making its recycling more feasible [28]. The suggested techniques may easily generate a range of flat and curved product shapes from scrap prepreg, and demonstrator components back up this assertion [29].

The parts include a curved section, a prototype RSRF prosthetic ankle/foot, and a typical hat stiffening panel [30]. Additional demonstration parts, including tubes, flat panels, and sandwich panels, have been created in the lab of United Arab Emirates University under the Department of Chemical and Petroleum Engineering [31,32].

Scrap prepreg can be used to make structural design parts that can be used to construct larger structures. Thin and thick panels, cylinders, sandwich structures with honeycomb or foam cores, hat stiffeners, and even scrap prepreg with a variety of types of fiber and resins, such as glass, carbon, and Kevlar fibers with diverse composites, have all been combined in the same part [33]. The components based on CF/epoxy have maximum expected application in the construction, both indoor and outdoor furnishing, and building structures due to their durability against humidity and the least vulnerability to insect invasion [34]. The CF/epoxy’s components are durable in the presence of humidity, and it is a great material for interior and external furnishings, as well as a basic building element because it has a low insect invasion. Container ships, which are currently composed of denser materials such as aluminum, worn steel, and wood, could benefit from the usage of prepreg trash [35].

Green environmental legislation and worldwide regulations have compelled researchers to investigate several strategies for recycling CFRP trash. However, this is difficult because the heat-stable matrices of CFRP do not melt with heating. The techniques other than heating are considered less viable due to their complex procedures. Thermo-mechanical recycling, therefore, is investigated by many researchers all around the globe as the optimum method in such a scenario [36]. Thermoset polymers are employed as matrices in structural CFRPs, accounting for about 80% of polymer composites. Mechanical strength, durability, chemical, and thermal resistance, and dimensional stability are all advantages of this material. However, because processed thermoset polymers are crosslinked, recovering the fibers is challenging as they cannot be easily heated, shaped, processed, or re-crosslinked following treatment [37]. Recycling CFRP trash reduces greenhouse-gas emissions associated with CFRP waste while also providing an inexpensive option to make high-value carbon fibers. By 2030, 6000–8000 business-related aircraft will have reached the end of their useful lives. As a result, there is an urgent need to create effective and sustainable waste management and recycling techniques for CFRPs [38].

#### 1.2.1. Recycling Techniques

CFRP trash is disposed of and burned at landfills. Different techniques such as incineration, laser-based burning, etc., are employed by different public and private sectors according to the quantity of disposal, resources available, and the applications desired [39]. Several nations have adopted landfill taxes in an attempt to reduce trash disposal, as well as material recycling, such as CFRP waste. CFRP waste recycling entails not only recycling carbon fiber but also using recovered carbon fibers (rCFs) in the manufacturing of new material. Therefore, the quality alongside the quantity of the recovered or recycled carbon fiber is required as an important parameter not only for the process of recycling but also for the further application of the recycled product [40]. 

**Figure 3:**
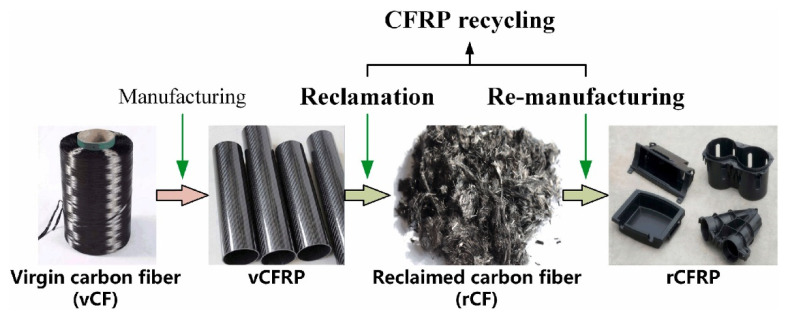
Recycling Process from Virgin Carbon Fiber to CFRP.

According to an article published in 2005 by Keiji Ogi, “As far as the authors know, products employing recycled CFRP have not been disclosed although some research may be undertaken in laboratories. As a result, no recycling system for CFRP wastes has been built around the globe thus far.” However, several uses have been developed since that time to properly utilize CFRP waste. Generally, recycling CFRP is divided into two stages: first, reclamation of carbon fibers from CFRP waste, then production of rCF reinforced polymer (rCFRP) as demonstrated in Figure 3 [41,42].

#### 1.2.2. Mechanical Method

Mechanical recycling is the most common method of recycling CFRP [43]. This approach consists of multiple procedures for reducing waste quantity. Initially, the CFRP is chopped to a size of 50–100 mm, and then additional grinding is employed to provide a range of properties ranging from ash to fibrous [44,45]. Damage to processing equipment caused by CFRP recycling procedures raises recycling operating costs, reducing the economic margin of recycling products. The mechanical procedure is risk-free and may be finished at room temperature. This method can hold debris up to 50 mm and attain 50–65% of the tensile strength of fresh carbon fibers. The most serious risk for this method is the dust generated by the recycling mechanism [46,47,48,49,50,51,52,53,54,55,56,57].

Fused Filament Fabrication (FFF) is considered one of the latest and most effective Additive Manufacturing techniques with high expectations of sustainable manufacturing, but the mechanical properties of polymers manufactured using this method are considerably lower than those manufactured by the traditional methods. Therefore, mechanical methods of recycling can be used for such processes [58].

#### 1.2.3. Pyrolysis

Pyrolysis is the thermal breakdown of polymers in the absence of oxygen in the atmosphere (300 °C to 800 °C), allowing the fibers to rebound with a high modulus. A greater temperature (around 1000 °C) can be employed, but this would result in mechanical property degradation. Carbon fibers derived from pyrolysis retained 90 percent of their original mechanical strength, according to studies. Pyrolysis is also used for the investigation of the types of binding materials used in the polymeric materials available in modern paints as implemented in the research of multi-analytical analysis of the “Orange Car Crash” study [59]. Furthermore, the polymeric framework has the potential to be utilized as oil droplets of liquid hydrocarbons. The pyrolysis method is recognized as a long-term CFRP recycling process [60].

#### 1.2.4. Chemical Method

Several solvents are used in the chemical process to break down polymer resin and separate it from carbon fibers. Chemical recycling can recover high-quality carbon-6 fibers with a tensile strength of 98.9%. According to the latest stats, recycling CFRP with the chemical technique consumes 38.4 MJ/kg of energy, which is nearly equivalent to 10–30% of the total energy required to manufacture new fibers. Although [61,62,63,64,65] the chemical approach is feasible, the treatment temperature, solvents, and equipment have negative environmental consequences.

The objective of this investigation is to handle two different wastes, PLA and different carbon fiber leftovers, domestically to produce and characterize composite material as an attempt to find a solution to the increasing industrial wastes. Different experimental tests have been carried out to study the impact of using carbon waste as reinforcement to the PLA on the properties of the processed composites.

## 2. Materials and Methods

**Figure 4:**
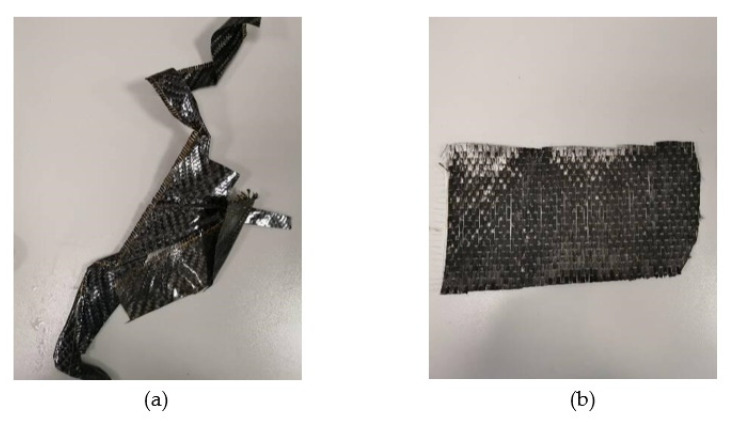
The original shape of the samples (**a**) CFRP, (**b**) CF.

**Figure 5:**
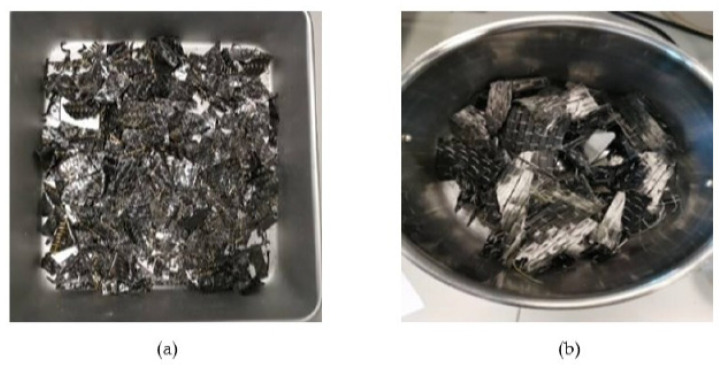
Samples after the cutting (**a**) CFRP, (**b**) CF.

**Figure 6:**
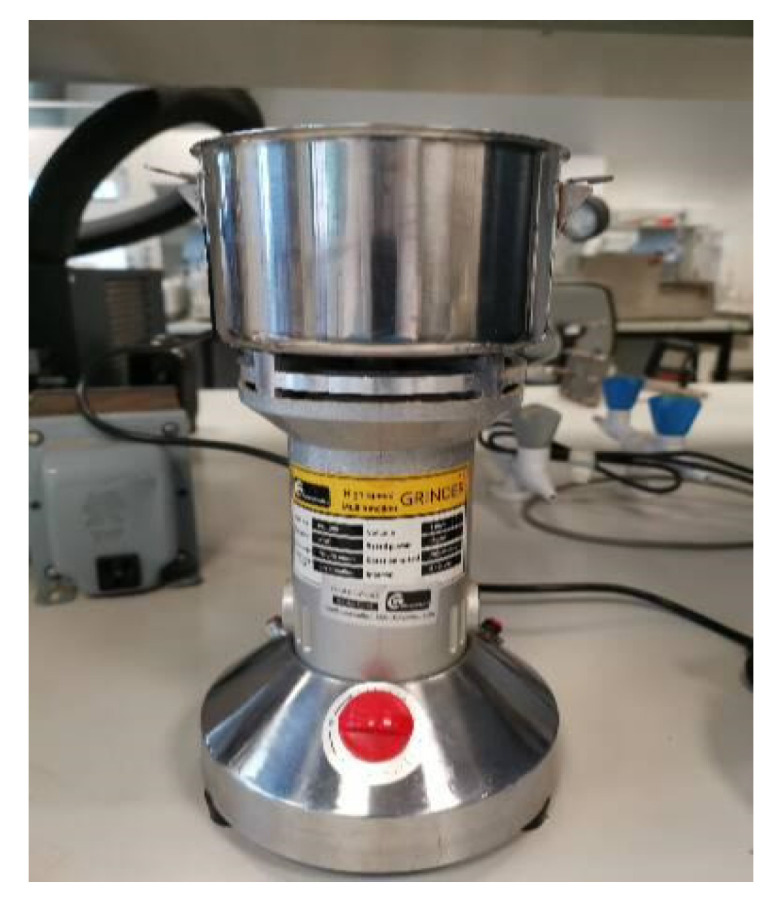
High speed grinder.

**Figure 7:**
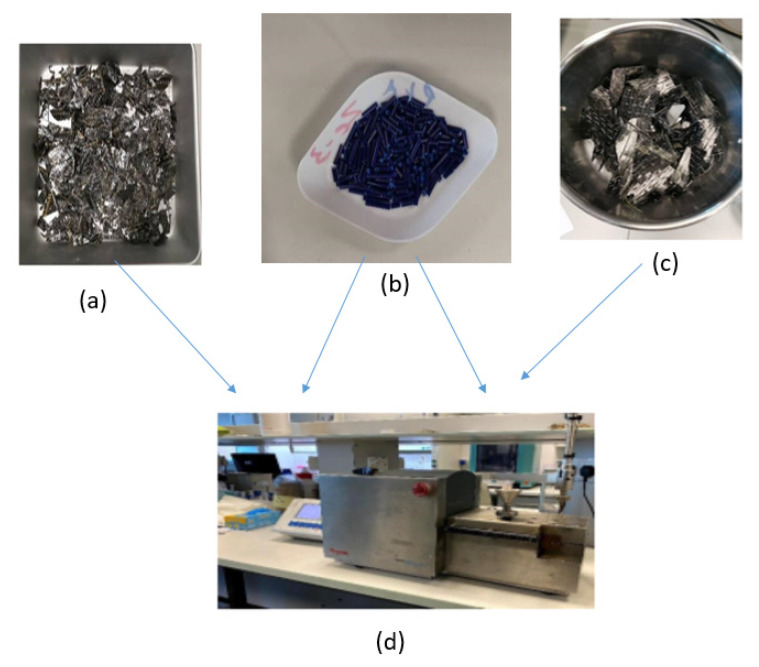
Samples of (**a**) CFRP, (**b**) PLA, (**c**) CF, (**d**) twin-screw extruder.

Assorted leftover PLA from 3D printing was collected from the prototyping labs of the university, sorted based on color, and then shredded. Two kinds of materials have been used: CF-Prepreg sheets (CFRP) and carbon fiber sheets (CF), as shown in Figure 4. Scissors were used to cut both materials into small pieces as shown in Figure 5. In the shredding process, a strong DIY shredding with stainless steel blades as illustrated in Figure 6 was utilized to break down the residual waste material into little bits that may be used to shred hard plastics and prepreg carbon fiber waste, whereas twin extruder (Figure 7) is used to process the material [65,66,67,68].

The shredded material was subsequently broken down into smaller bits with a heavy-duty mixer designed for hardwoods, as the shredded material did not include shredder residue [65]. Since the plastic waste came from lab sources, the initial stage of the shredding process was carefully performed to break down the residual waste material. The process of shredding is important and equally critical so that the Crystal-kinetic properties are linked with the process [69]. Therefore, this process is carried out with utmost care and control. The color of the original samples is purplish black. A schematic diagram of the formation process of the waste PLA/CF composite is shown in the figure below.

**Figure 8:**
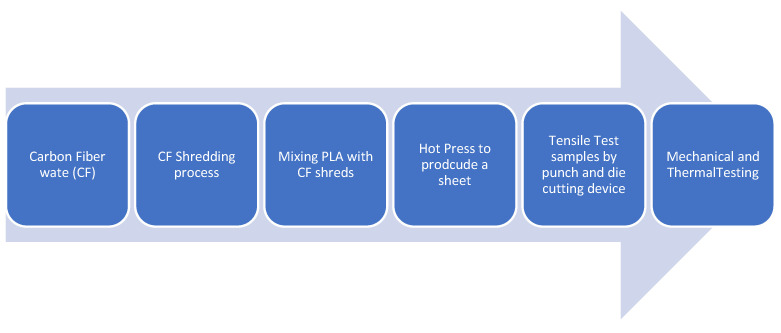
Formation of PLA/CF composite.

Different percentages of both materials (CF and CFRP) mixed with PLA polymer were melt-blended into a composite material in a twin-screw extruder (MiniLab Rheomex CTW5, HAAKE, Karlsruhe, Germany). The melt blending technique used a closed-loop cycle with a rotating speed of 140 rpm and a temperature of 190 °C for 5 min. The blended material was extruded via a valve at the extruder’s outlet to collect the composite material for the compression molding stage after the closed-loop cycle. Figure 8 shows the material preparation and extrusion process.

The extruded sample was cut into small pieces and thermally compressed under 5000 psi and at the same extrusion temperature and time using a Carver’s press (CarverTM Lab Presses, 1569 Morris Street, PO Box 298 Wabash, IN 46992-0298 USA). This procedure was followed for each polymer’s extruded samples with the required filling ratio. The procedure was followed for the extruded samples so that the dimensional coherency would be achieved for the composite samples made by the compression process, which will produce thin sheets that will be examined to investigate the mechanical and thermal properties to explore the potential for using different waste carbon fibers to enhance the properties of the recycled PLA. This analysis was helpful for the determination of a proper understanding of material performance under mechanical and thermal characterization [70]. The samples obtained as a result of this technique were thin sheets of composite material that were examined for mechanical and thermal properties. Many researchers have evaluated the effectiveness of the recycling process experimentally to demonstrate the potential of using fiber waste as a reinforcement to enhance recycled plastics [71,72,73,74,75,76,77,78,79,80,81].

Furthermore, several problems occurred during the experiments. The first obstacle was with the high-speed grinder used to turn the samples into powder. The high-speed grinder operation duration is one minute, while the remainder of the cooling down time is five minutes, resulting in a lengthy wait. However, to save time, two high-speed grinders were utilized at the same time, one for each specimen. The other difficulty was with the Hot melt extrusion (HME) process because five distinct samples were produced separately in a sequential process which could lead to a possible discrepancy in the properties of the tested samples. The cleaning time was around 30 min, while the operation time was approximately 10 min.

**Figure 9:**
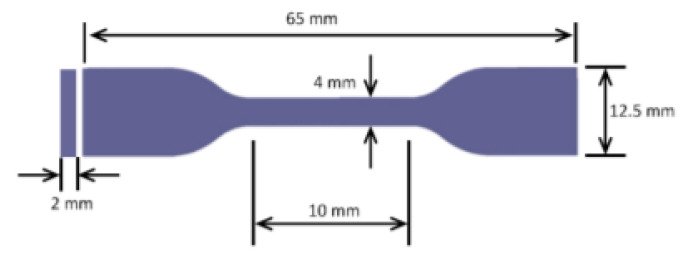
Dimensions of the Specimen.

To investigate the influence of CFRP and CF reinforcement on polymer/composite samples, researchers used a universal testing machine (UTM, Shimadzu, Kyoto, Japan). The tensile test samples were prepared using the thin composite sheets resulting from the compression molding stage, as shown in Figure 9, according to the American Society for Testing and Materials (ASTM)-D 638 [82] guidelines. This was to determine the mechanical modulus of elasticity, yield strength, ultimate strength, and strain at the failure point.

**Figure 10:**
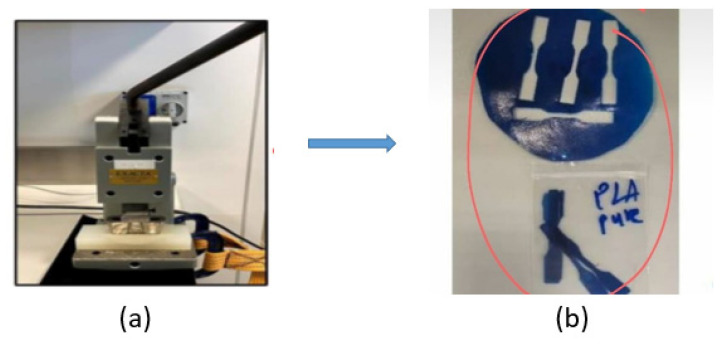
(**a**) Blanking machine, (**b**) Dumbbell shape samples after blanking.

**Figure 11:**
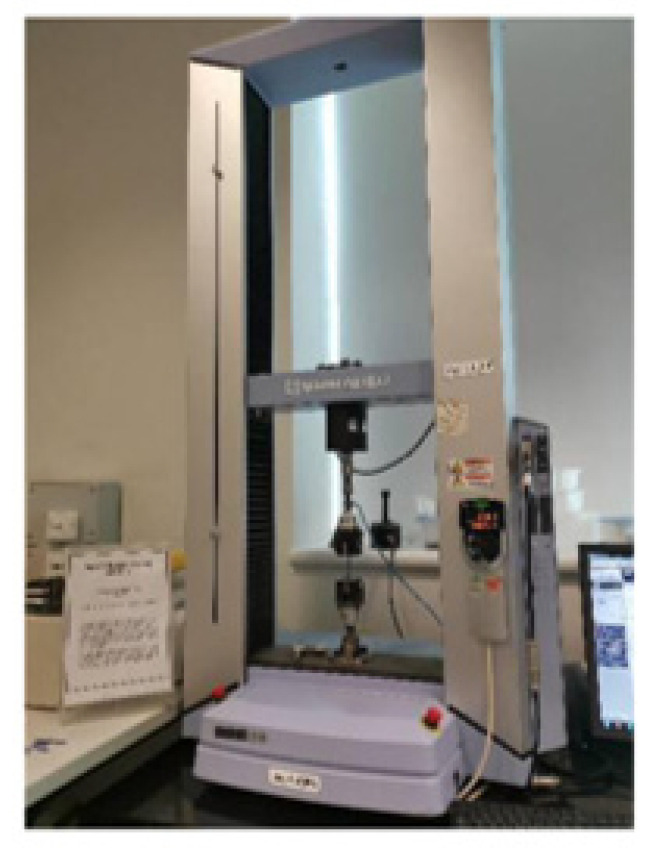
Tensile testing machine with a sample.

A unique manual blanking machine was used to acquire the specific dimensions (Exacta Model-JFP, Bengaluru, India). The blanking machine was used to make dumbbell-shaped tensile test specimens as shown in Figure 10a. Each pair of composite samples’ dimensions (thickness (t)) are listed in Table 1. The accompanying mechanical properties were investigated using a 10 kN load cell with a 5-mm/min crosshead movement. The blanking technique is depicted in Figure 10b. Each sample had three weights, each with three trails, for a total of 15 tests to achieve higher accuracy, which are shown as standard deviations (SDs) in the results plots. The average gauge length and width of the samples are 17.02 mm and 2.69 mm, respectively. The result of the tensile test was obtained. The tensile testing machine is shown in Figure 11.

An XRD test was conducted on untreated, NaOH treated, and silane-acetone treated fillers to examine the impact of the treatment on the crystalline structure. The equipment used was a Malvern Panalytical X-ray diffractometer. The clearance between the punch and the die is a typical problem in cutting the samples that is supposed to be adjusted based on the sample thickness. Instead of using a standard cutting device, the clearance is constant and cannot be changed. This would affect the sample edge quality, which would impact the failure of the samples during the tensile test [83,84]. As a result, the sheared edges may be altered. Accordingly, hard surfaces emerge, altering the sample’s mechanical properties and perhaps leading to unpredictable collapse. Stress concentrations at the sample’s end frequently impact the mechanical behavior of the sheets.

During the blanking process, the pressure applied to the punch and die may generate stress distributions, which can affect the geometry difference between a specific burr size and the intersecting surface [85]. The extruded composites were cut into little bits due to the limitations of the extruder, which creates restricted quantities of the combinations. As a result, the sheared edges may be altered, resulting in the formation of rough surfaces that change the mechanical characteristics of the sample and may lead to inconsistent failure.

### 2.1. Fourier Transform (FTIR)

The infrared test is used to evaluate the presence of the functional groups, CF and CFRP. The chemical reaction occurred as a result of the inclusion of the filler. The Fourier Transform Infrared Spectroscopy (FTIR) is used to confine the successful coupling between the polypropylene matrix and the filler [82]. The FT/IR-4700 from JASCO was used for the test.

### 2.2. XRD

To investigate the effect of pretreatment on the crystalline structure, XRD tests were performed on an untreated filler, NaOH treated filler, and silane-acetone treatment bonding agent. The Malvern Analytical X-ray diffract meter was utilized.

### 2.3. TGA

TGA testing is used to determine thermal deterioration and thermal stability. TGA Q500 from TA Instruments was used for the test. The apparatus was loaded with samples weighing 5 to 10 mg, and the temperature was increased from 40 to 800 °C. TGA data may be shown using two graphs: weight % versus temperature and derivative weight versus temperature.

## 3. Results and Discussion

Two kinds of composite materials—CF/PLA and CFRP/PLA—have been produced to investigate different properties, such as thermogravimetric analysis (TGA), X-ray diffraction (XRD), and Fourier-transformed infrared spectroscopy (FTIR), as well as the tensile testing machine. Basically, CFRP sheets and carbon fiber sheets (CF) were used to produce thin layers of the composite blends mixed with PLA, and many tensile specimens were punched from each sheet using a commercial dog-bone cutting machine. The Mitutoyo thickness gauge (Model 547-526S) was used to determine the thickness of the thin film due to the high resolution (0.001 mm) and accuracy (0.0002). In this study, the average readings along the gage length of the sample were considered [86].

### 3.1. Mechanical Characterization

**Figure 12:**
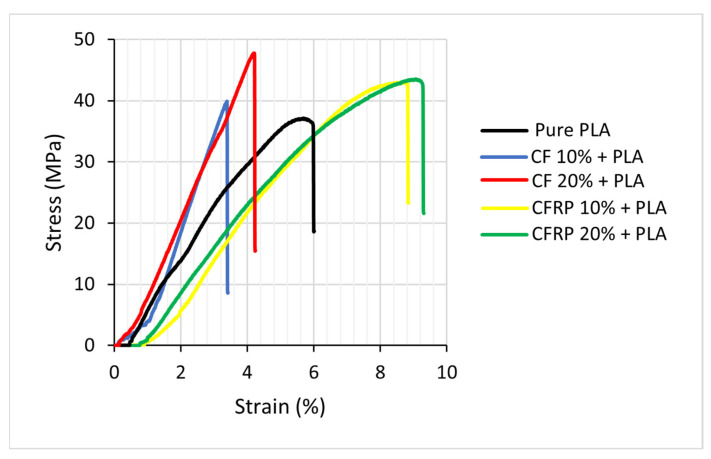
Stress-strain curve for the recycled PLA and four recycled composites.

A sample of the stress–strain curves for the five composites investigated in this study is depicted in Figure 12, where the detailed values are listed in Table 2. In general, it has been observed that the tensile test experimentations revealed that carbon fiber enhanced the mechanical properties of the recycled PLA. However, the CF composite at 20% demonstrated significantly higher ultimate strength, with around 10% more than the CFRP for the same reinforcement percentage. Still, for the 10%, the CFRP is 7% higher than the CF, whereas the ductility for the CFRP composites possesses higher values due to the expired impregnated epoxy in the prepreg leftover pieces [87,88,89]. A reduction of 18.4% in the ultimate tensile strength of the recycled PLA concerning the commercial PLA 3D Printing Ultimaker filament properties (UTS = 45.6 MPa) has been estimated [90].

Table 2 illustrates the mechanical properties of the experimental work conducted in this research associated with the standard deviations.

Figure 12 shows that the stress–strain properties of the recycled PLA properties are lower than the reinforced Carbon fiber/PLA composite due to the fact the reinforcements enhance the properties of the pure recycled PLA [91,92].

The parameter that characterizes materials’ resistance to deformations is the modulus of the elasticity, i.e., stiffness, which is the extent to which an object resists deformation in response to an applied force [93]. There are several ways to estimate stiffness from experimental data. It can be either the slope of the stress–strain curve at any specified stress or strain (tangent stiffness or tangent modulus), or the slope of the straight line connecting the origin of the stress–strain curve with another point on the curve (secant stiffness or secant modulus). Among all the tangent moduli, moreover, the tangent modulus at the origin (initial stiffness) is of particular significance. This value is associated with the behavior of the material for low load values, a behavior that is linear elastic for most brittle materials. Therefore, the tangent modulus at the origin is equivalent to Young’s modulus in the elastic (reversible) deformation regime. Usually, the secant modulus is, instead, a percentage of Young’s modulus and describes the stiffness of a material in the inelastic region of the stress–strain diagram. To evaluate the tangent modulus at the origin correctly, it is of fundamental importance to have reliable experimental data for low load values.

**Figure 13:**
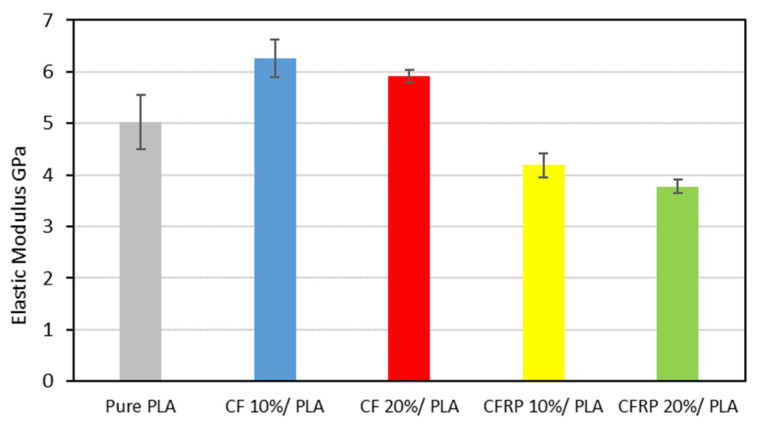
Elastic Modulus of the blends.

Figure 13 demonstrates the elastic properties of the produced composites. It is observed that samples produced with 10% CF have the highest elastic modulus among the blends due to the impact of the fiber reinforcement, which is 33% higher than the 10% CFRP. Compared with the recycled PLA, the elastic modulus increased by 18% at 20% CF, whereas a reduction of 24% to 20% in the CFRP samples was observed. A similar trend could also be observed [94] that increased the Young’s Modulus value up to a certain level of content of fibers in the matrix.

**Figure 14:**
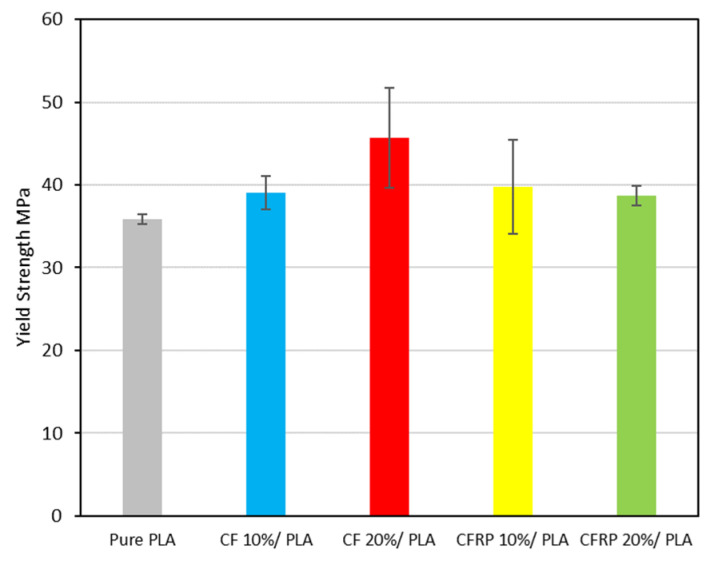
Yield strength for the samples.

Figure 14 shows the yield strengths for the various samples and indicates that for the 20% CF with PLA, the resulting yield strength is the highest, while 20% CFRP + PLA leads to the lowest value of the yield strength. The overall trend for the (10% CFRP, 20% CFRP, 10% CF, and 20% CF) samples showed a rise in the yield strength due to the contribution of the carbon fibers to the PLA properties’ mixing. Moreover, the yield strength of pure recycled PLA shows a drop of 27% with respect to the commercial PLA filament. In general, the yield strength is estimated at a specific strain rate of 0.2%, which is the most standard method, commonly referred to as proof stress.

**Figure 15:**
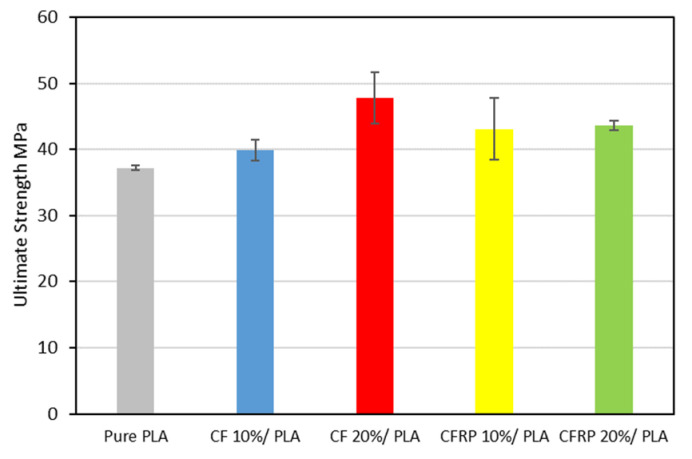
Ultimate Tensile Strength.

Figure 15 presents the Ultimate Strength of the produced samples. It has been shown that the ultimate strength of the CF/PLA bends increased with the increase of the CF contents, with an increase from 7% to 28% for the CFRP at 10% and 20% contents, respectively, in comparison to the recycled PLA [95]. On the other hand, it is shown that the CFRP/PLA ultimate strength increased slightly concerning the recycled PLA, but it has a lower level than the CF/PLA blends, which is ascribed to the presence of the expired impregnated epoxy, as clarified before. The slight increase in the ultimate strength is estimated to be from 15% to 17% for the 10% and 20% CFRP contents, respectively.

**Figure 16:**
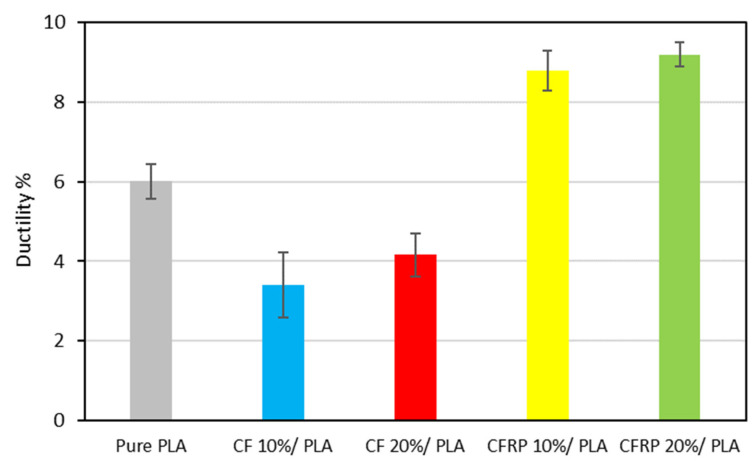
Strain at the failure point.

The trends of the ductility of the composite blends, i.e., the strains recorded at the point of failure during the tensile testing procedure, have been graphically plotted in Figure 16. It has been observed that the recycled PLA ductility has a higher strain (6.01%) at the failure point in comparison with the commercial PLA filament (5.2%), with an increase of around 15% in ductility due to the degradation of the recycled properties during the recycling process [95]. Moreover, clear evidence illustrates that the CF/PLA composite suffered from a remarkable drop in ductility ranging from 43% to 30% for the 10% and the 20% CF reinforcement, respectively, which is attributed to the impact of the carbon fiber reinforcement. On the contrary, the CFRP/PLA composites exhibited a significant increase in ductility from 46% to 53% for the 10% and the 20% CFRP reinforcement, respectively. The reason behind this observation is the expired impregnated epoxy of the leftover prepreg waste that caused degradation of the composite matrix [79].

The standard deviation (Equation (1)) is estimated by calculating the arithmetic mean of the obtained values.

(1) $$S=\sqrt{(\sum X^{2}-n\beta )/\left(n-1\right)}$$

where *S* is the estimated standard deviation, *X* is the value of a single reading, *n* is the number of measurements, and *β* = is the set of observations’ arithmetic means.

### 3.2. XRD, FTIR, and TGA Analysis

**Figure 17:**
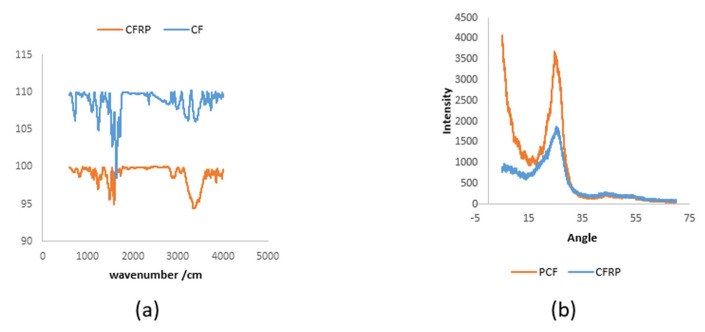
(**a**) FITR spectra of CFRP and CF; (**b**) XRD spectra of CFRP and CF.

Figure 17 shows the XRD and FTIR results for both CFRP and CF. It is clear that the scattering peak is at an angle of 2θ = 25.5 [96,97]. In the FTIR test, CFRP and CF exhibit nearly identical peaks. There are several distinctive absorption peaks at about 717, 1238, 1637, 3230, and 3432 cm^−1^. Of these peaks, the peak at 717 cm^−1^ was qualified to the bending vibrations of methylene (CH_2_^−^), and 1238 cm^−1^ is the peak from the C-F bands. The peak at 1637 cm^−1^ was attributed to stretching vibrations of the C = C groups. The peak at 3230 cm^−1^ and 3432 cm^−1^ was ascribed to the stretching vibrations of the OH groups.

**Figure 18:**
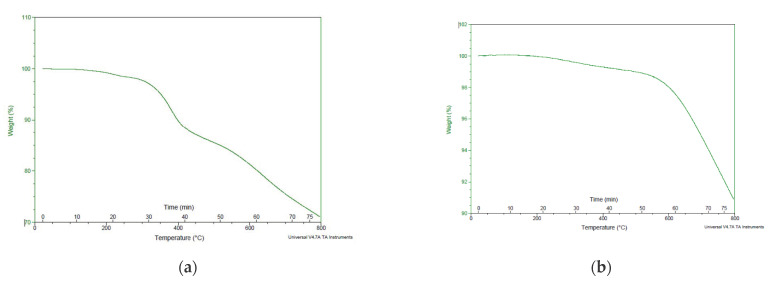
The TGA result for (**a**) CFRP (**b**) CF.

Figure 18 shows two graphs: (a) is the TGA for CFRP, which indicates that they began to lose weight after 300 °C, and (b) is the TGA for CF, which indicates that they began to lose weight after 300 °C. At the highest temperature, which is 800 °C, the sample loses 70% of its weight. The CF outcome is more stable than the CFRP. At around 700 °C, the weight began to drop [98,99,100,101,102,103,104,105]. The PLA was resistant to a continuous transition heat due to lacking of contaminants. Furthermore, the addition of fillers has no significant effect on Tm. The modest changes in the curve forms are most likely related to the degree of crystallization for each test, suggesting that the composite material’s superior heat stability is preserved.

## 4. Conclusions

In this research, the adopted recycling method was proven practically to be a feasible process for the recycling of the leftover carbon fiber and the uncured CFRP prepreg material that can be utilized in various nonstructural end-products. The experimental methodology adopted for the research included the combination of different types of carbon fiber CF and the CFRP prepreg mixed with waste PLA, hence the conversion of the leftover prepreg material into intermediate products such as CF-Prepreg 10% with PLA, CF-Prepreg 20% with PLA, CF10% with PLA, CF20% with PLA, and pure PLA, followed by the production of various end products using these intermediate products through the hot extrusion and compression molding processes.

The low cost of the feedstock and simple, effective, and rapid processing methods should result in low-cost commercial end products that retain the strong performance for which composites are known. Furthermore, the experiment provides a practical, low-cost method through which the components made from waste prepreg can be made in aesthetically good physical forms, with a smooth and unique surface finish due to the random orientation and arrangement of the fibers. We studied the characteristics and performance of scrap prepreg-based laminates under various starting material states and processing circumstances and drew several conclusions that have been summarized as follows:

1. The experimental investigation revealed the negative impact of the expired impregnated epoxy of the CFRP prepreg leftover scrape that may affect the mechanical properties of the produced composite, even though the properties of the CFRP/PLA composites in general showed better properties than the recycled PLA.
2. For the modulus of elasticity, the CF/PLA composite at 20% has higher properties than the CFRP at 20%—specifically, the elastic modulus is 65% and 56% higher for the CF and the CFRP, respectively, in comparison to the PLA reference value.
3. The yield strength of the 20% CF is the highest, whereas the 20% CFRP is the lowest.
4. Carbon fiber in general enhanced the ultimate strength of the carbon/PLA blends, starting from 7% at 10% CF to 28% at 20% CF, and 15% at 10% CFRP to 17% at 20%.
5. For the ductility, the recycled PLA showed a higher level of 15% compared to the commercial PLA filament. For the CF/PLA, the CF has an opposite impact on the ductility: it decreased the strain at the failure from 43% to 30% for 10% and 20% CF, respectively. In contrast, CFRP has been attributed to uplifting the strain at failure from 46% to 53% at the 10% and the 20% CFRP, respectively.

Producing a composite for recycled PLA and the carbon leftover waste demonstrates promising properties that could be used for various applications employing different ratios of the mixing parameters that can be successfully carried out with the formed material compositions. Although the economic impact of the treatment on the process has yet to be determined, it appears that this research will serve as the starting point for future work on the recovery of chemical compounds from CFRP polymer resins.

## Figures and Tables

**Table 1:** Dimensions of the prepared tensile test specimens.

Samples	Length mm	Width mm	Thickness mm
CF-Prepreg 10% + PLA	17.02	2.69	0.28
CF 10% + PLA			0.20
CF-Prepreg 20% + PLA			0.24
CF 20% + PLA			0.26
Pure-PLA			0.27

**Table 2:** The mechanical properties of the recycled Carbon/PLA composites with standard deviation (SD).

Material ID	Elastic Modulus		Yield Strength		Ultimate Strength		Ductility	
	GPa		MPa		MPa		%	
	Value	SD	Value	SD	Value	SD	Value	SD
Pure PLA	5.02	±0.53	35.83	±0.60	37.20	±0.40	6.01	±0.44
CF 10%/PLA	6.26	±0.36	39.07	±1.97	39.90	±1.60	3.40	±0.82
CF 20%/PLA	5.91	±0.12	45.68	±6.00	47.81	±3.90	4.16	±0.55
CFRP 10%/PLA	4.19	±0.23	39.77	±5.70	43.07	±4.70	8.80	±0.50
CFRP 20%/PLA	3.78	±0.13	38.73	±1.21	43.58	±0.70	9.20	±0.31
